# Ultra-High Hydrostatic Pressure Pretreatment on White Que Zui Tea: Chemical Constituents, Antioxidant, Cytoprotective, and Anti-Inflammatory Activities

**DOI:** 10.3390/foods12030628

**Published:** 2023-02-01

**Authors:** Xiaoyu Zhang, Mengcheng Li, Li Zhen, Yudan Wang, Yifen Wang, Yuyue Qin, Zhihong Zhang, Tianrui Zhao, Jianxin Cao, Yaping Liu, Guiguang Cheng

**Affiliations:** 1 The Faculty of Food Science and Engineering, Kunming University of Science and Technology, Kunming 650500, China; 2 Kunming Institute of Zoology, Chinese Academy of Sciences, Kunming 650000, China; 3 The Faculty of Food and Bioengineering, Jiangsu University, Zhenjiang 212013, China

**Keywords:** *Lyonia ovalifolia*, ultra-high hydrostatic pressure pretreatment, UHPLC-MS/MS analysis, antioxidant activity, anti-inflammatory activity

## Abstract

Herbal tea has numerous biological activities and exhibits broad benefits for human health. In China, the flower buds of *Lyonia ovalifolia* are traditionally processed as herbal tea, namely White Que Zui tea (WQT). This study was aimed to evaluate the effect of ultra-high hydrostatic pressure (UHHP) pretreatment on the chemical constituents and biological activities of free, esterified, and insoluble-bound phenolic fractions from WQT. A total of 327 chemical constituents were identified by a quasi-targeted metabolomics analysis. UHHP pretreatment extremely inhibited reactive oxygen species (ROS) production and cell apoptosis in H_2_O_2_-induced HepG2 cells, and it increased the activities of intracellular antioxidant enzymes (SOD and CAT) and GSH content in different phenolic fractions from WQT. In addition, after UHHP pretreatment, the anti-inflammatory effects of different phenolic fractions from WQT were improved by inhibiting the production of nitric oxide (NO) and pro-inflammatory cytokines (TNF-α, IL-6, and IL-1*β*) in LPS-induced RAW264.7 cells. Thus, the UHHP method might be a potential pretreatment strategy for improving the bioavailability of phytochemicals from natural plants.

## 1. Introduction

Tea is the most widely used beverage around the world with a long history of consumption. Usually, tea can be grouped into six categories, containing oolong tea, green tea, white tea, black tea, yellow tea, and dark tea according to different processing techniques [1]. Generally, these teas are mainly made by the fresh leaves of *Camellia sinensis* (tea plant). Furthermore, herbal teas derived from the leaves and flowers of medicinal or edible plants play an important role in our daily life with various biological activities [2]. Recently, increasing investigations were performed on herbal teas such as Shi Ya tea [3], Rooibos tea [4], Tibetan tea [5], Mate tea [6], and so on. They have anticancer, neuroprotective, anti-inflammatory, antitumor, hepatoprotective, and antiviral activities [7,8]. In addition, phenolic compounds constitute the important chemical composition in herbal teas, and they are potent bioactive agents in the human body.

Plants are rich in natural products including flavonoids, lignans, saponins, steroids, and polysaccharides. Some of these have potent immunomodulatory, anti-inflammatory, antioxidant, hypoglycemic, hypolipidemia, and anticancer activities, and they have potential in the treatment of inflammation-related diseases, metabolic disorders, and cancers [9,10,11,12,13,14]. Generally, phenolic compounds exist in plants in three forms, which are free, esterified, and insoluble-bound phenolics [15,16]. Free and esterified phenolic fractions are relatively easy to extract with a high extraction rate, whereas the insoluble-bound phenolic fraction is bound to biomacromolecules, resulting in a low extraction rate [17]. In recent years, solvent extraction, supercritical fluid extraction, microwave-assisted extraction [18], ultra-high hydrostatic pressure technology (UHHP) [16], and ultrasound-assisted extraction [19] have been developed to increase the extraction rate of polyphenols, especially insoluble-bound phenolics. Moreover, a recent study has found that UHHP combined with ultrasound-assisted extraction technology can significantly enhance the extraction efficiency of insoluble-bound phenolic compounds [16].

The flower buds of *L. ovalifolia*, also known as White Que Zui tea (WQT) in Yunnan province, are used as a popular herbal tea. In addition, the flowers of *L. ovalifolia* have been also used to treat swelling, dermatitis, and intestinal inflammation as a Traditional Chinese Medicine [20]. A previous study reported that *L. ovalifolia* presented terpenoids, lignans, and flavonoids [21]. To date, the phytochemical investigation on *L. ovalifolia* is limited, and its biological assessment has not been studied yet.

This paper aimed to investigate the effects of UHHP pretreatment on the chemical constituents of six phenolic fractions and compared their antioxidant activities, cytoprotective effects, and anti-inflammatory activities before and after UHHP pretreatment.

## 2. Materials and Methods

### 2.1. Chemicals

DCFH-DA and an Annexin V-FITC/PI apoptosis kit were purchased from Beijing 4A Biotech Co., Ltd. (Beijing, China). DPPH, ABTS, TPTZ, MTT, and LPS were purchased from Sigma-Aldrich (Shanghai, China). SOD, CAT, and GSH kits were purchased from Nanjing Jiancheng (Nanjing, China). FBS, DMEM, streptomycin, and penicillin were purchased from Gibco (Grand Island, NY, USA). ELISA kits for human TNF-α, IL-6, and IL-1*β* were obtained from (Lianke) Biotech (Hangzhou, China).

### 2.2. Materials and Treatment

The WQT was collected from Wuding country in Yunnan Province, China. Firstly, the WQT was crushed into powder form by a grinder and divided into two groups. One group was used directly for the extraction of different phenolic fractions. The other group was transferred into a sealed vacuum package. Subsequently, the powder was treated at 500 MPa by using ultra-high hydrostatic pressure equipment (HHP-600, Baotou Kefa High Pressure Technology Limited Company, Baotou, China) for 10 min, followed by ultrasound-assisted extraction with a methanol aqueous solution, and the collected filtrate was freeze-dried.

### 2.3. Extraction of Different Phenolic Fractions

The free, esterified, and insoluble-bound phenolic fractions in the normal and UHHP-pretreated WQT were carried out according to our previous study [16]. In brief, 10 g of the WQT powder was degreased three times with petroleum ether (1:10, *w*/*v*). For further sample extraction, the powder was ultrasonically extracted with 70% methanol aqueous solution three times (30 min each time) at room temperature. Afterwards, the supernatant was collected after filtrating with a Buchner funnel and concentrated to remove methanol by a rotary evaporator (IKA, Germany). The remaining aqueous solvent was adjusted to pH 2.0 with 6 mol/L HCl. Free fractions were extracted with ethyl acetate-ether five times (1:1, v/*v*). The free phenolic fractions (FP) were obtained by freeze-drying after the supernatant was concentrated. The 4 mol/L NaOH (1:1, v/*v*) was used to hydrolyze the remaining aqueous phase for 4 h at room temperature and then acidified to pH 2.0 with 6 mol/L HCl. By fractionation with ethyl acetate-ether, the esterified phenolic fraction (EP) was obtained after collecting and concentrating the organic phase. The remaining solid residue was hydrolyzed for 4 h with 4 mol/L NaOH and filtrated with the Buchner funnel. The filtrate was adjusted to pH 2.0 with 6 mol/L HCl, and then ethyl acetate-ether was used to extract the insoluble-bound phenolic fractions (IBP). The samples were preserved at 4 °C for further study.

### 2.4. UHPLC-MS/MS Analysis of WQT

The chemical constituents in WQT were performed by using the UHPLC-MS/MS quasi-targeted metabolomics analysis. The FP, EP, IBP, UFP, UEP, and UIBP after liquid nitrogen grinding were treated in methanol, vortexed and shook, and let stand for 5 min in an ice bath. The solution was centrifuged at 15,000 g for 20 min, and then the supernatant was subjected to the UHPLC-MS/MS analysis after the filtration by a 0.22 μm microporous membrane.

The chromatographic conditions were the following: column (Xselect HSS T3, 2.5 μm, 2.1 × 150 mm); ultrapure water and acetonitrile (mobile phase A and B containing 0.1% methanoic acid) were utilized for the analysis. The gradient elution procedure was as follows: 0–2 min, 5–95% B; 2–15 min, 95–100% B; 15–17 min, 100% B; 17.01–20 min, 5% B. The column temperature was set at 50 °C, the flow rate was 0.4 mL/min, and the injection volume was 2 μL, respectively. Typical ion source parameters were as follows: curtain gas pressure: 35 psi; ionspray voltage: −4500 V; temperature: 550 °C; ion source gas 1 and 2 pressure: 60 psi; DP: ± 100 V. Based on the NovoDB database (novogene database), the sample was identified using the multiple reaction monitoring mode (MRM) in order to obtain the mass spectra of several samples. The SCIEX OS V1.4 software was used to integrate and calibrate the mass spectra and then output the relative molecular masses, retention times, peak areas, and identification results. 

### 2.5. Determination of the Content of Total Phenolics (TPC) and Total Flavonoids (TFC)

The TPC was measured by the Folin-Ciocalteu (FC) method using the previously reported literature [22]. All samples were dissolved in 80% methanol aqueous solution. In short, the sample solution (1.0 mL, 1.0 mg/mL) and the FC reagent (0.5 mL) were reacted for 1 min, and then distilled water (7.0 mL) and the Na_2_NO_3_ solution (1.5 mL, 20% *m*/*v*) were added for further incubation in a heated water bath (70 °C). The absorbance of all samples was recorded at 765 nm in a Spectra-Max M5 microplate reader (Molecular Devices, Sunnyvale, CA,USA). The result was calculated as milligrams of gallic acid equivalent (GAE)/g dry extract.

The TFC was determined by using the method described previously [23]. In short, the sample solution (1.0 mL), the ethanol aqueous solution (1.5 mL, 60% *m*/*v*), and the NaNO_2_ solution (0.15 mL, 5% *m*/*v*) were successively added to the Eppendorf tubes and reacted for 8 min after uniformly mixing. Afterwards, the mixture was reacted with Al (NO_3_)_3_ (10% *m*/*v*, 0.15 mL), NaOH (4% *m*/*v*, 2.0 mL), and the ethanol solution (60% *m*/*v*, 6.7 mL) in turn at room temperature. After 12 min, the absorbance was determined immediately at 510 nm. The TFC was expressed as mg of the rutin equivalent per gram of extract (mg RE/g).

### 2.6. Evaluation of Antioxidant Activity by DPPH, ABTS, and FRAP Assays

#### 2.6.1. DPPH Assay

The antioxidant activities of different phenolic fractions in WQT were analyzed by researching their scavenge capacity of the DPPH radical based on a previous method after a slight adjustment [24]. The sample solutions (0.5 mL) at concentrations of 12.5, 25, 50, 100, and 200 μg/mL were mixed with the DPPH reagent (0.1 mmol, 2 mL) and placed in the dark environment. The absorbance value under 517 nm was determined after 30 min. Following this formula:

(A control − A sample)/A control × 100 (1)

the DPPH radicals scavenging activity (%) was computed.

#### 2.6.2. ABTS Antioxidant Assay

The ABTS antioxidant assay was measured based on the approach described previously [25]. The sample solutions (0.5 mL) of different concentrations were treated with the ABTS solution (4.0 mL) in a water bath (30 °C) for 6 min, and afterwards the absorbance of different phenolic fractions was measured under 734 nm. The calculation method was similar to the DPPH assay.

#### 2.6.3. Determination of Ferric-Reducing Antioxidant Power (FRAP)

The FRAP value of WQT was performed in terms of the reported method [26]. The 300 mM acetic acid solution (pH = 3.6), 20 mM FeCl_3_, and 10 mM TPTZ were mixed together (10:1:1, *v/v/v*) and heated at 30 °C to obtain the FRAP solution. The preheated FRAP solution (4.5 mL) was reacted with 0.5 mL of the sample solution, which was then treated for 10 min at 30 °C. The absorbance value at 593 nm was then measured.

### 2.7. Intracellular Antioxidant Assay

#### 2.7.1. Cytotoxic Test

DMEM supplemented with 1% penicillin-streptomycin and 10% FBS was used to culture human HepG2 cells. The cells were then cultured at 37 °C in a cell incubator with 5% CO_2_.

A cytotoxicity test was carried out by the MTT method [27]. Briefly, HepG2 cells (1 × 10^5^ cells/mL) were cultured in a 96-well plate. After 24 h, the cells were incubated containing the samples of different concentrations. Next, after 20 h, the cell culture medium was removed, the MTT solution was applied to each well for 4 h at a concentration of 0.5 mg/mL. Then, DMSO was used to dissolve the formazan. The absorbance at 570 nm was measured. 

#### 2.7.2. Measurement of Reactive Oxygen Species

Based on the previous method [28], the intracellular ROS production was determined. In 6-well plates, HepG2 cells were cultured at a density of 1 × 10^5^ cells per well. Afterwards, the samples at a concentration of 50 μg/mL were cocultured with the cells for 24 h, and V_C_ (10 μg/mL) was used as a positive control. H_2_O_2_ was added to all groups excluding the control group with a final concentration of 1.0 mmol/L for 6 h. Whereafter, the cells were washed thrice with PBS, and then treated for 30 min with DCFH-DA (10 μM) at 37 °C in the dark. By using flow cytometry (Guava^®^ simple Cyte 6-2L, Millipore, Billerica, USA), the ROS level was immediately determined.

#### 2.7.3. Evaluation of SOD, CAT Activities, and GSH Content

To analyze the effects of different phenolic fractions on CAT, GSH, and SOD expression, HepG2 cells were cultured as per Section 2.7.1. After centrifugation for 10 min, the cells were collected and homogenized in the phosphate buffer. The intracellular antioxidant enzyme activities were measured by using commercially available kits. 

### 2.8. Determination of Cellular Apoptosis Assay

A quantitative analysis of cell apoptosis was determined by using the Annexin V-FITC/PI apoptosis kit and flow cytometry. The specific experimental method was similar to the method of the ROS experiment. The cells were stained with Annexin V-FITC (5.0 μL) and 10.0 μL PI (20.0 μg/mL), then incubated in the dark environment. The cellular apoptosis assay was immediately determined by flow cytometer.

### 2.9. Determination of NO Level and Pro-Inflammatory Cytokines (TNF-α, IL-6, and IL-1β) Assay

The RAW264.7 cells were purchased from the Cell Bank, Kunming Institute of Zoology. The cell culture and cytotoxicity tests were performed as per Section 2.7.1. Briefly, the cells (1 × 10^5^ cells/mL) were seeded into 24-well plates for 24 h. FP, EP, IBP, UFP, UEP, UIBP, and DXM were treated for 4 h. Subsequently, all groups except the model group were stimulated with LPS (1.0 μg/mL) for 20 h. Finally, the cell supernatant was collected, and the contents of NO, TNF-α, IL-6, and IL-1*β* were measured based on the instructions of the commercial kit [29].

### 2.10. Statistical Analysis

All tests were performed in triplicate and the experimental data were expressed as the mean ± SD, with *p* < 0.05 indicating statistical significance. A one-way ANOVA and Tukey’s test were used to evaluate the data analysis and principal component analysis using Origin 9.0 software (Origin Lab, Northampton, MA, USA).

## 3. Results and Discussion

### 3.1. The Chemical Constituents in WQT

Chemical constituents from WQT were studied by the UHPLC-MS/MS quasi-targeted metabolomics analysis. The total ion chromatograms (TICs) of the different phenolic fractions in WQT are shown in Figure S1 (supplementary material Figure S1). A total of 327 constituents were determined under the negative ESI mode (supplementary material Table S1) based on the NovoDB database (novogene database), including 39 phenylpropanoids, 91 flavonoids, 36 phenols, 60 carbohydrates, 24 terpenoids, 2 alkaloids, 2 quinones, 13 benzoic acids, and 60 organic acids. As shown in Figure S1 and Table S1, protocatechuic acid, gallic acid, and rutin were the abundant phenolic compounds in the FP, EP, and IBP, respectively, according to their relative higher peak heights and areas. After the UHHP pretreatment, the relative contents of chemical constituents of UFP, UEP, and UIBP were significantly increased. For example, the content of gallic acid in UEP was 1.33 times that of EP, and the content of rutin in UIBP was nearly two-fold higher than that in IBP. This phenomenon might be related to the principle of UHHP on phytochemical extraction. UHHP pretreatment could destroy the cell structure and block the binding bind between the secondary metabolites (especially phenolic acids and flavonoids) and biomacromolecules (lignin, polysaccharides, or proteins) [30]. These findings were the same as our previously reported data that UHHP could increase the content of phenolic compounds [16].

### 3.2. The Contents of Total Phenolics (TPC) and Total Flavonoids (TFC) in Different Phenolic Fractions from WQT

**Figure 1:**
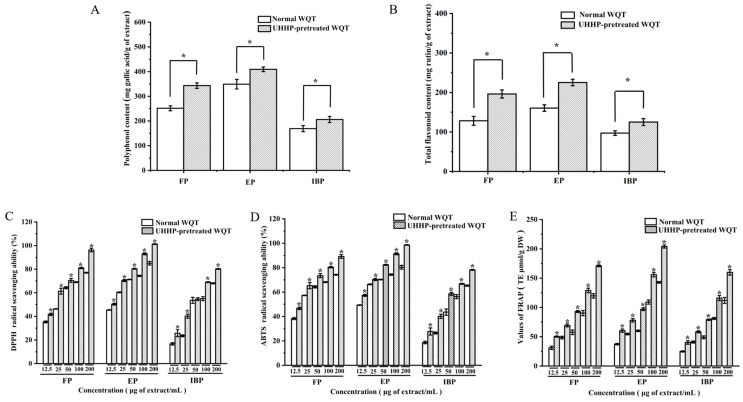
The contents of total phenolics and total flavonoids as well as their antioxidant activities of free, esterified, and insoluble-bound phenolic fractions from normal and UHHP-pretreated White Que Zui tea (WQT). (**A**): Total phenolic content; (**B**): total flavonoid content; (**C**): DPPH radical scavenging activity; (**D**): ABTS radical scavenging ability; (**E**): iron reduction antioxidant capacity (FRAP). All the values are expressed as mean ± SD (n = 3). “*” means significant differences between the same fractions from the normal and UHHP-pretreated WQT (*p* < 0.05). FP and UFP: free phenolic fraction from normal and UHHP-pretreated WQT; EP and UEP: esterified phenolic fraction from normal and UHHP-pretreated WQT; IBP and UIBP: insoluble-bound phenolic fraction from normal and UHHP-pretreated WQT.

The TPC and TFC in FP, EP, IBP, UFP, UEP, and UIBP were presented in Figure 1A,B. Among the FP, EP, and IBP, the TPC and TFC values of EP were the highest with 349.01 ± 19.36 mg GAE/g extract and 160.41 ± 8.3 mg RE/g extract (*p* < 0.05), respectively. However, the TPC and TFC values of UEP increased to 409.27 ± 9.36 mg GAE/g extract and 225.3 ± 8.3 mg RE/g extract after UHHP pretreatment, respectively. The values of TPC and TFC of FP also increased from 251.38 ± 10.2 to 343.4 ± 11.2 mg GAE/g extract, and from 128.3 ± 11.3 to 196.2 ± 10.3 mg RE/g extract after UHHP treatment, respectively. Nevertheless, IBP showed the lowest levels of TPC and TFC with 169.14 ± 12.3 mg GAE/g extract and 97.1 ± 5.7 mg RE/g extract, respectively (*p* < 0.05), which were almost half of those of EP. UIBP possessed low TPC and TFC values with 206 ± 12.4 mg GAE/g extract and 125.1 ± 8.7 mg RE/g extract (*p* < 0.05), respectively. Previous studies indicated that the peel of araticum fruit (*Annona crassiflora* Mart) principally involved esterified phenolic fractions [31]. In addition, it is noteworthy that the TPC and TFC of UEP were about two times those of the EP. Therefore, UHHP pretreatment could enhance the extraction yield and biological accessibility of phenolic compounds regardless of the free, esterified, and bound phenolics. This result was in agreement with the above chemical constituents.

### 3.3. Antioxidant Activities of Different Phenolic Fractions from WQT

Phenolic compounds exist widely in the plant kingdom, with simple or complex structures, and have been shown to have potent antioxidant activities in vivo or in vitro experiments [32]. Therefore, DPPH, ABTS, and FRAP assays were used in the current study to evaluate the antioxidant capacities of different phenolic fractions in WQT with or without UHHP pretreatment.

#### 3.3.1. DPPH Radical Scavenging Abilities of Different Phenolic Fractions from WQT

The determination of DPPH radical scavenging ability is applied generally to evaluate the scavenging ability of phytochemicals [33]. The DPPH scavenging activities of different phenolic fractions from WQT were positively related with the concentration of phenolic compounds. EP possessed the strongest DPPH radical scavenging activity in normal phenolic fractions of WQT as shown in Figure 1C. With concentrations ranging from 12.5 to 200 μg/mL, its DPPH radical scavenging rates increased from 45 to 85%. Moreover, the IC_50_ values of FP, EP, and IBP were 28.57 ± 1.34 μg/mL, 14.79 ± 2.30 μg/mL, and 48.77 ± 2.54 μg/mL in the DPPH radical scavenging assay, respectively. After UHHP pretreatment, the DPPH scavenging abilities of UFP, UEP, and UIBP were significantly enhanced (*p* < 0.05), and the IC_50_ values of them were 17.86 ± 0.34 μg/mL, 12.39 ± 1.3 μg/mL, and 40.45 ± 1.54 μg/mL, respectively. In addition, it is noteworthy that UEP and UFP on DPPH radical scavenging activities reached almost 100% at a concentration of 200 μg/mL. The Pearson correlation coefficients between antioxidant activities, and TPC and TFC values were investigated in the present study. The IC_50_ values of DPPH radical scavenging activities were negatively correlated with TPC and TFC contents in different phenolic fractions from WQT (r = −0.97 and −0.88, respectively, *p* < 0.05). 

Phenolics and flavonoids might be the main contributor of WQT to the antioxidant capacities. Moreover, phenolic acids are regarded as effective hydrogen donors because of their characteristic easily ionized carboxyl groups; the number of hydroxyl groups was positively correlated with antioxidant capacity [34]. Gallic acid had four hydroxyl groups in its molecular structure [35]. Compared to other phenolic fractions, UEP had the strongest ability to scavenge free radicals. This result might be related to the highest content of gallic acid in UEP.

#### 3.3.2. ABTS Radical Scavenging Abilities of Different Phenolic Fractions from WQT

As demonstrated in Figure 1D, the ABTS radical scavenging abilities of different phenolic fractions from WQT increased with increasing concentrations. In particular, EP possessed the highest ABTS scavenging ability, regardless of UHHP pretreatment. FP, EP, and IBP had IC_50_ values of 20.9 ± 1.4, 12.7 ± 2.2, and 79.51 ± 2.5 μg/mL, respectively. Furthermore, the IC_50_ values of UFP, UEP, and UIBP in WQT were 13.25 ± 1.34 μg/mL, 10.59 ± 1.36 μg/mL, and 39.36 ± 1.4 μg/mL, respectively. Thus, UHHP pretreatment could promote the ABTS scavenging abilities of different phenolic fractions in WQT. The significant linear correlations (r = −0.85 and −0.78, *p* < 0.05) were surveyed between the contents of TPC, TFC, and the IC_50_ values of ABTS radical scavenging activities in FP, EP, IBP, UFP, UEP, and UIBP. These results indicated that phenolic constituents in WQT had a close relationship with the ABTS radical scavenging activity.

#### 3.3.3. FRAP Evaluation of Different Phenolic Fractions from WQT

The FRAP method is an efficient and sensitive method to detect the antioxidant capacity of phytochemicals [36]. The FRAP values of UFP, UEP, and UIBP (Figure 1E) were extremely higher than those of FP, EP, and IBP in a concentration-dependent manner (*p* < 0.05), especially UEP. Moreover, this result was positively correlated with the TPC and TFC (r = 0.82 and 0.94, *p* < 0.05). The above data indicated that the FRAP values of the different phenolic fractions in WQT increased linearly with increasing TPC and TFC values. Therefore, the antioxidant capacity may be related to the contents of TPC or TFC.

### 3.4. Intracellular Antioxidant Activities of Different Phenolic Fractions from WQT

#### 3.4.1. Inhibitory Effect on ROS Production

ROS are produced during the breathing process of living organisms and are crucial for biological processes such as cell proliferation, apoptosis, and signal transduction [37]. H_2_O_2_ could readily penetrate the cell membrane, producing a large number of free radicals that attack the mitochondrial membrane, resulting in excessive intracellular ROS production [38]. Excessive ROS could cause oxidative stress to induce cell and tissue damage, thereby resulting in genomic instability, cell death, or tumorigenesis [39]. 

**Figure 2:**
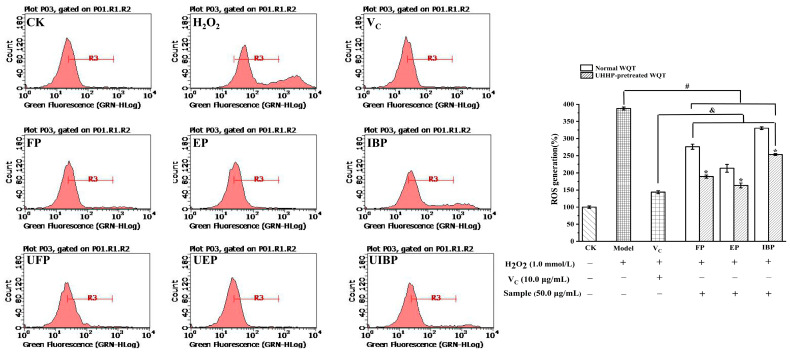
The inhibitory effects of intracellular ROS of the free, esterified, and insoluble-bound phenolic fractions from the normal and UHHP-pretreated White Que Zui tea (WQT) in H_2_O_2_-induced HepG2 cells. “*” indicated significant differences in the same fractions between the normal and UHHP-pretreated WQT (*p* < 0.05); “#” indicated significant differences between the H_2_O_2_ group and the sample groups (*p* < 0.05); “&” indicated significant differences between the V_C_ group and the sample groups (*p* < 0.05). All the values were expressed as mean ± SD (n = 3). FP and UFP: free phenolic fraction from normal and UHHP-pretreated WQT; EP and UEP: esterified phenolic fraction from normal and UHHP-pretreated WQT; IBP and UIBP: insoluble-bound phenolic fraction from normal and UHHP-pretreated WQT.

In this study, the inhibitory effects of different phenolic fractions from WQT on ROS production in H_2_O_2_-induced HepG2 cells were explored by flow cytometry. The cytotoxicity experiment manifested that all samples were non-toxic on HepG2 cells at a concentration up to 100 μg/mL. Therefore, 50 μg/mL in this study was selected for further investigation. The intracellular ROS level is presented in Figure 2: the relative amount of ROS in the model group increased to 387.84 ± 4.27%, which was almost three times higher than that of the control group. The six phenolic fractions could efficiently suppress intracellular ROS generation. The relative ROS contents of the UFP, UEP, and UIBP were significantly lower than those of FP, EP, and IBP (*p* < 0.05), which indicated that UHHP pretreatment of WQT could observably suppress ROS production. Interestingly, UEP (163.25 ± 7.09%) had the strongest inhibitory effect activity, and its effect was essentially similar to that of the positive group (V_C_ with 143.6 ± 4.45%). Additionally, the ROS levels in the samples showed a close statistical correlation with their TPC (r = −0.94) and TFC (r = −0.96). The relative content of phenolic compounds increased after UHHP pretreatment. Proto-catechuic acid, gallic acid, and rutin as the major phenolic constituents in FP, EP, and IBP, had significant antioxidant capacity [40,41,42]. Thus, the phenolics and relative content of phytochemicals may be the main influencing factors on the ROS inhibition [22].

#### 3.4.2. Effects of Different Phenolics Fractions on Cellular SOD, CAT Activities, and GSH Level

SOD, CAT, and GSH are significant parts in the cellular antioxidant enzyme system. They can mitigate oxidative damage in H_2_O_2_-induced HepG2 cells and maintain the dynamic balance of oxidative stress in the antioxidant defense system [43]. Intracellular antioxidant enzymes will scavenge free radicals as endogenous antioxidants when oxidative stress occurs [44]. The SOD, CAT activities, and GSH level were determined to evaluate whether all different phenolic fractions from WQT had a positive effect on endogenous enzymatic activities in HepG2 cells induced by H_2_O_2_.

**Figure 3:**
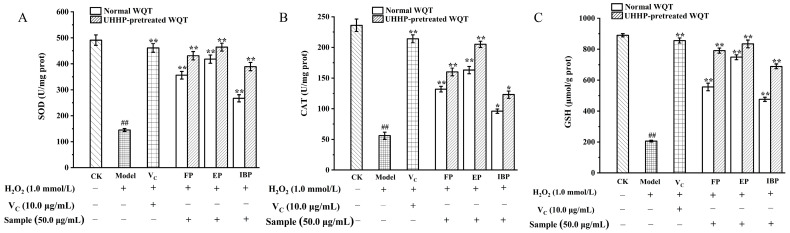
The effects of the free, esterified, and insoluble-bound phenolic fractions from the normal and UHHP-pretreated White Que Zui tea (WQT) on SOD (**A**), CAT (**B**), and GSH (**C**) expressions. * *p* < 0.05 compared to the model group; ** *p* < 0.01 compared to the model group; ## *p* < 0.01 compared to the control group. All the values are expressed as mean ± SD (n = 3). FP and UFP: free phenolic fraction from normal and UHHP-pretreated WQT; EP and UEP: esterified phenolic fraction from normal and UHHP-pretreated WQT; IBP and UIBP: insoluble-bound phenolic fraction from normal and UHHP-pretreated WQT.

In this study, the SOD and CAT activities as well as the GSH level dramatically decreased in the model group in comparison to the control group (*p* < 0.01) (Figure 3). Figure 3 indicates that UEP had the highest antioxidant ability with 464 ± 15.2 U/mg, 205 ± 5.22 U/mg, and 834 ± 25.2 U/mg of SOD, CAT, and GSH, respectively, followed by EP (418 ± 16.1, 163 ± 6.05, and 748 ± 16.05 U/mg, respectively), while IBP showed the lowest SOD, CAT activities, and GSH level. Furthermore, the cytoprotective activity of UEP was similar to that of the positive group (V_C_). The result was similar to the above ROS results. Thus, WQT could protect HepG2 cells from H_2_O_2_-induced oxidative stress not only by scavenging intracellular ROS, but also by enhancing the defense system of antioxidant enzymes.

### 3.5. Inhibitory Effect on H_2_O_2_-Induced Cell Apoptosis of Different Phenolic Fractions from WQT

**Figure 4:**
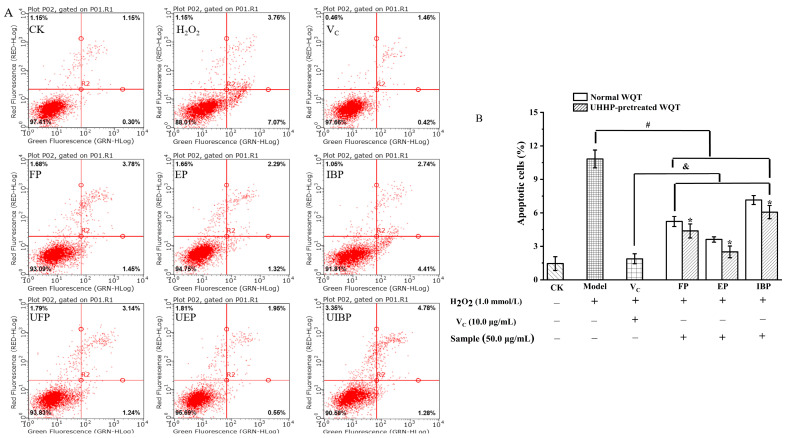
The inhibitory effects of the free, esterified, and insoluble-bound phenolic fractions from the normal and UHHP-pretreated White Que Zui tea (WQT) on apoptosis in H_2_O_2_-induced HepG2 cells. (**A**): flow cytometry analysis; (**B**): the apoptotic cell percentages of different groups. “*” indicated significant differences in the same fractions between the normal and UHHP-pretreated WQT (*p* < 0.05); “#” indicated significant differences between the H_2_O_2_ group and the sample groups (*p* < 0.05); “&” indicated significant differences between the V_C_ group and the sample groups (*p* < 0.05). All the values are expressed as mean ± SD (n = 3). FP and UFP: free phenolic fraction from normal and UHHP-pretreated WQT; EP and UEP: esterified phenolic fraction from normal and UHHP-pretreated WQT; IBP and UIBP: insoluble-bound phenolic fraction from normal and UHHP-pretreated WQT.

Cell apoptosis, as a normal physiologic procedure, is defined as the independent and orderly death by genes’ regulation to maintain a stable homeostasis. However, abnormal cell apoptosis will induce inflammatory diseases and autoimmune disorders [38]. In the present study, the cell apoptosis results of different phenolic fractions in WQT were measured by flow cytometer and are described in Figure 4. After pretreatment with 1.0 mM H_2_O_2_, the cell apoptosis rate in the H_2_O_2_-treated group (10.83 ± 0.8%) was significantly higher than that of the control group (1.45 ± 0.62%). However, compared to the model group, the cell apoptosis of all samples was remarkably suppressed (*p* < 0.05), especially after UHHP treatment. UEP displayed the strongest anti-apoptosis effect, followed by EP, UFP, FP, and UIBP, whereas IBP had the lowest activity (*p* < 0.05). Interestingly, the cell apoptosis rate of the UEP was close to that of the positive group. In six phenolic fractions, there was a highly negative correlation between the cell apoptosis and their TPC (r = −0.98) and TFC (r = −0.91). These results suggested that the phenolic compounds of WQT may be responsible for its cytoprotective effects. The previous literature also showed that UHHP pretreatment increased the anti-apoptotic effects of the different phenolic fractions of mango leaves [16].

### 3.6. Anti-Inflammatory Activities of Different Phenolic Fractions from WQT

**Figure 5:**
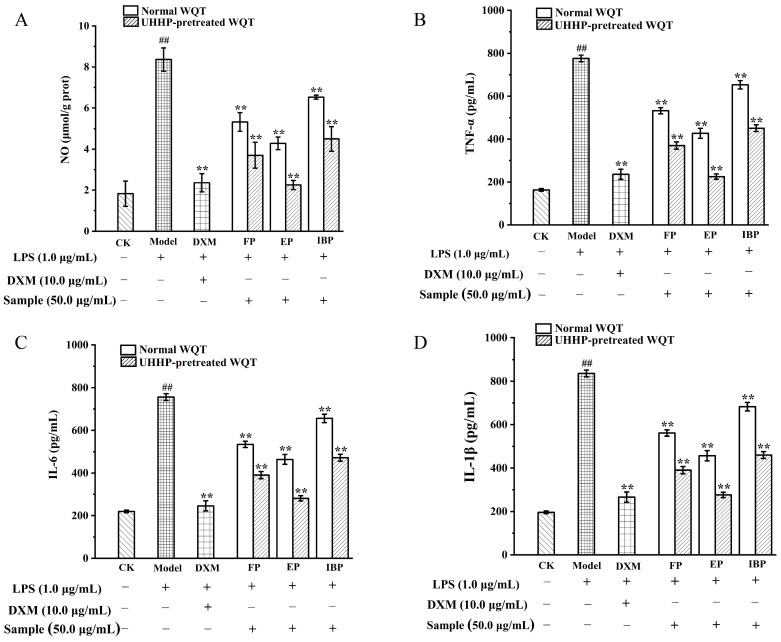
The inhibiting effects of NO and pro-inflammatory factors (TNF-α, IL -6, and IL-1*β*) produced by LPS-induced RAW264.7 cells from the normal and UHHP-pretreated White Que Zui tea (WQT). (**A**): NO content; (**B**): TNF-α; (**C**): IL -6; (**D**): IL-1*β*. ** *p* < 0.01 compared to the model group; ## *p* < 0.01 compared to the control group. All the values are expressed as mean ± SD (n = 3). FP and UFP: free phenolic fraction from normal and UHHP-pretreated WQT; EP and UEP: esterified phenolic fraction from normal and UHHP-pretreated WQT; IBP and UIBP: insoluble-bound phenolic fraction from normal and UHHP-pretreated WQT.

Inflammation, an important component of immunopathogenesis, responds to pathological conditions through the production of NO and pro-inflammatory cytokines [45]. During inflammatory diseases, macrophages overproduce mediators that cause damage to cells, ultimately leading to the severe clinical symptoms of immune disorders [46]. The different phenolic fractions from WQT were determined for their anti-inflammatory activities on LPS-induced RAW264.7 cells. As shown in Figure 5A, the expression of NO was greatly increased in the model group compared to the control group (*p* < 0.01). The release of NO was inhibited after the intervention of different phenolic fractions from WQT, especially after UHHP pretreatment. In addition, UEP had the strongest inhibitory effect on NO expression, which was equal to the effect of the positive group (DXM). The expression of NO in the UEP decreased by two-fold compared to that of EP.

As described in Figure 5B–D, the model group markedly upregulated the expression of inflammatory cytokines including TNF-α, IL-6, and IL-1*β* (*p* < 0.01), which were suppressed after the treatment with all different phenolic fractions. Furthermore, UEP displayed the best inhibitory effect on inflammatory cytokines, which was comparable to that of DXM. Therefore, WQT exhibited anti-inflammatory effects by reducing the excessive expression of pro-inflammatory cytokines and NO. UHHP pretreatment can disrupt the cell wall, resulting in rapid penetration of the solvent into the cell, and facilitating the elution of the compounds [15]. The phenolic content increased significantly after UHHP treatment. The anti-inflammatory effect observed may be due to the synergistic effect of the phenolic compounds identified in the extract [47]. Furthermore, a previous study has also revealed that the extracts with high polyphenol content could inhibit the NO level and the production of pro-inflammatory cytokines in LPS-induced RAW264.7 cells [48].

### 3.7. PCA Analysis

**Figure 6:**
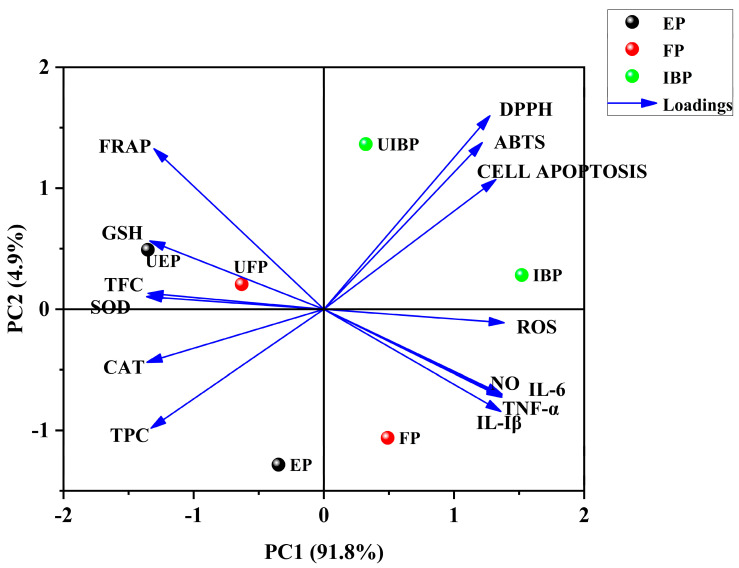
PCA analysis on TPC, TFC, antioxidant activities, cytoprotective effects, and anti-inflammatory activities. FP and UFP: free phenolic fraction from normal or UHHP-pretreated White Que Zui tea (WQT); EP and UEP: esterified phenolic fraction from normal or UHHP-pretreated WQT; IBP and UIBP: insoluble-bound phenolic fraction from normal or UHHP-pretreated WQT.

A principal component analysis (PCA) was used to analyze the variation of experimental data on the biological activities of FP, EP, IBP, UFP, UEP, and UIBP from WQT. Figure 6 shows the total variation accounted for 96.7%, of which PC1 accounted for 91.8% and PC2 accounted for 4.9%. The upper left quadrant of PC1 included UFP and UEP that possessed higher flavonoid contents and FRAP value. Meanwhile, they were opposite to the inhibitory effect of intracellular ROS and cell apoptosis, indicating that UEP and UFP had better cytoprotective effects. Furthermore, UIBP and IBP presented in the upper right quadrant of PC1, demonstrating poor ABTS and DPPH radical scavenging activities. In addition, EP and UEP showed strong inhibitory effects on NO expression and pro-inflammatory cytokines’ production (TNF-α, IL-6, and IL-1*β*), and had the highest TPC and TFC contents.

## 4. Conclusions

In this paper, UHHP pretreatment extremely enhanced the TPC, TFC, antioxidant activities, cytoprotective effects, and anti-inflammatory activities of FP, EP, IBP, UFP, UEP, and UIBP from WQT. A total of 327 chemical constituents were identified by a quasi-targeted metabolomics analysis, and WQT was mainly rich in flavonoids and phenolic compounds. Different phenolic fractions from WQT exhibited potential antioxidant activities by scavenging DPPH and ABTS radicals, and they had significant cytoprotective effects by inhibiting oxidative stress damage and cell apoptosis in H_2_O_2_-induced HepG2 cells. Moreover, six phenolic fractions from WQT also inhibited NO and pro-inflammatory cytokine (TNF-α, IL-6, and IL-1*β*) expressions in LPS-stimulated RAW264.7 cells. Particularly, UEP had the highest phenolic and flavonoid contents, and exhibited the strongest antioxidant activities, cytoprotective effects, and anti-inflammatory activities. Based on the above experimental results, UHHP could enhance the extraction rate of phytochemicals and bioactivities of WQT, which provide an effective value for further utilization and development of WQT in functional food applications.

## Data Availability

Data will be made available on request.

## Supplementary Materials

The following supporting information can be downloaded at: https://www.mdpi.com/article/10.3390/foods12030628/s1, Figure S1: The total ion chromatograms (TICs) from the normal and UHHP-pretreated White Que Zui tea (WQT); Table S1: The compounds identified in different fractions from White Que Zui tea (WQT) by quasi-targeted metabolomics analysis.

## Abbreviations

ABTS	2,2′-azino-bis (3-ethylbenzothiazoline-6-sulfonic acid)
CAT	Catalase
DCFH-DA	2′,7′-dichlorofluorescin diacetate
DMEM	dulbecco′s modified eagle′s medium
DPPH	2-diphenyl-1-picrylhydrazyl radical
DXM	Dexamethasone
FBS	fetal bovine serum
GSH	Glutathione
IL-1*β*	interleukin-1β
IL-6	interleukin-6
LPS	Lipopolysaccharide
MTT	3-(4,5-dimethylthiazol-2-yl)-2,5-diphenylte-trazolium bromide
SOD	superoxide dismutase
TNF-α	tumor necrosis factor-α
TPTZ	1,3,5-tri(2-pyridyl)-2,4,6-triazine
