# Supplementary material for: Ultra-High Hydrostatic Pressure Pretreatment on White Que Zui Tea: Chemical Constituents, Antioxidant, Cytoprotective, and Anti-Inflammatory Activities
Source: Foods. 2023 Feb 1;12(3):628. doi: 10.3390/foods12030628 (PMC9914134; doi:10.3390/foods12030628)
Supplement: Supplementary file 1 [file foods-12-00628-s001.zip › foods-2108689-supplementary/supplementary materials/Supplementary Materials Table S1.pdf]

Table S1. The compounds identified in different fractions from White Que Zui tea (WQT) by quasi-targeted metabolomics analysis.

Peak	Name	Formula	Molecular Weight	RT [min]	Class	CAS	FP	EP	IBP	UFP	UEP	UIBP
1	Dimethylmalonic acid	$C_5H_8O_4$	132.042	0.67	Organic Acids	"595-46-0"	558400	3748000	1303000	847100	1661000	1975000
2	Afzelechin	$C_{15}H_{14}O_5$	274.084	0.68	Flavonoids	"2545-00-8"	113800	278100	57160	162300	59500	109900
3	2-O-Caffeoylmalic acid	$C_{13}H_{12}O_8$	296.053	0.69	Polyphenol	"53755-04-7"	25190	144300	39680	39540	28230	52060
4	6'-O-Feruloyl-D-sucrose	$C_{22}H_{30}O_{14}$	518.164	0.69	Polyphenol	"118230-77-6"	79780	562600	79630	287200	87060	145900
5	Oxalacetic acid	$C_4H_4O_5$	132.07	0.69	Organic Acids	"328-42-7"	458600	2416000	850500	668600	1248000	1366000
6	3-Phenylpropanoic acid	$C_9H_{10}O_2$	150.174	0.7	Phenylpropanoids	"501-52-0"	252300	228300	130800	308900	161400	291100
7	Kuwanon A	$C_{25}H_{24}O_6$	420.454	0.7	Flavonoids	"62949-77-3"	12450	246700	22130	21630	32940	50170
8	Melittoside	$C_{21}H_{32}O_{15}$	524.47	0.7	Terpenoids	"19467-03-9"	28281	221200	14940	1900	16330	27980
9	4-Hydroxy-2-Oxoglutaric Acid	$C_5H_6O_6$	162.098	0.7	Organic Acids	"1187-99-1"	661000	1510000	568600	1164000	512400	944900
10	Glutaric Acid	$C_5H_8O_4$	132.115	0.7	Organic Acids	"110-94-1"	667200	3870000	1318000	876700	1787000	2005000
11	alpha-Benzylsuccinic acid	$C_{11}H_{12}O_4$	208.211	0.71	Organic Acids	"884-33-3"	132300	1059000	324300	178700	354700	546200
12	2-Oxoadipic acid	$C_6H_8O_5$	160.037	0.72	Organic Acids	"3184-35-8"	902200	1428000	681200	1110000	541800	800000
13	6-Sialyllactose	$C_{23}H_{39}NO_{19}$	633.551	0.73	Carbohydrates	"35890-39-2"	12630	23950	8636	42540	11544	6885
14	D-Glucarate	$C_6H_{10}O_8$	210.139	0.82	Carbohydrates	"87-73-0"	618700	202200	57960	713500	169000	193500
15	3-Ureidopropionate	$C_4H_8N_2O_3$	132.053	0.82	Organic Acids	"462-88-4"	438600	42310	47156	811300	14730	140648
16	Ribose 5-phosphate	$C_5H_{11}O_8P$	230.02	0.84	Carbohydrates	"4300-28-1"	249800	23510	71550	384300	29040	70090
17	2,2-Dimethylsuccinic acid	$C_6H_{10}O_4$	146.058	0.84	Organic Acids	"597-43-3"	18300	5611	4009	24830	2392	6775
18	Sorbitol 6-phosphate	$C_6H_{15}O_9P$	262.045	0.86	Carbohydrates	"20479-58-7"	1076000	215800	88660	1074000	149200	37390
19	D-Glucose 6-phosphate	$C_6H_{13}O_9P$	260.03	0.86	Carbohydrates	"56-73-5"	12480000	558200	203400	20850000	889600	252400
20	D-Fructose 6-Phosphate	$C_6H_{13}O_9P$	260.136	0.86	Carbohydrates	"643-13-0"	35970000	1069000	498800	54380000	2121000	561800
21	O-Phosphorylethanolamine	$C_2H_8NO_4P$	141.063	0.86	Organic Acids	"1071-23-4"	506600	11250	11150	542700	62171	8025
22	Adipic acid	$C_6H_{10}O_4$	146.058	0.86	Organic Acids	"124-04-9"	84790	64300	111800	134700	115200	103200
23	D-Mannose 6-phosphate	$C_6H_{13}O_9P$	260.136	0.87	Carbohydrates	"723729-16-6"	624500	35420	8364	914500	56390	12280
24	alpha-D-Galactose 1-phosphate	$C_6H_{13}O_9P$	260.136	0.87	Carbohydrates	"2255-14-3"	24680000	600900	113000	26870000	669200	451200
25	D-Galactaric acid	$C_6H_{10}O_8$	210.038	0.87	Carbohydrates	"526-99-8"	2126000	933800	253900	2221000	683600	761600
26	D-Glucopyranose	$C_6H_{12}O_6$	180.156	0.87	Carbohydrates	"28905-12-6"	427900	1303000	258000	463800	1047000	432200
27	s7p	$C_7H_{13}BaO_{10}P$	425.473	0.87	Carbohydrates	"2646-35-7"	4392000	1443000	91610	4727000	1056000	214100
28	Ligustrazine	$C_8H_{12}N_2$	136.197	0.88	Alkaloids	"1124-11-4"	1622000	1017000	316800	1861000	1199000	321100
29	D-Fructose 1,6-bisphosphate	$C_6H_{14}O_{12}P_2$	340.116	0.88	Carbohydrates	"488-69-7"	121700	31600	78150	151400	84750	34730
30	Succinic anhydride	$C_4H_4O_3$	100.073	0.88	Organic Acids	"108-30-5"	23810000	8591000	603700	31940000	3713000	1099000
31	Cinnamyl acetate	$C_{11}H_{12}O_2$	176.21	0.89	Phenylpropanoids	"103-54-8"	229200	321800	49410	281700	351700	124500
32	L-Sorbose	$C_6H_{12}O_6$	180.16	0.89	Carbohydrates	"87-79-6"	326300	137200	39680	470000	159000	62420
33	D-Ribose 5-phophate	$C_5H_{11}O_8P$	230.11	0.89	Organic Acids	"207671-46-3"	1732000	106400	218100	2381000	186700	253300
34	D-Glucuronic acid	$C_6H_{10}O_7$	194.14	0.894	Carbohydrates	"6556-12-3"	201300	222400	179900	173000	260300	254600
35	Ribose 1-phosphate	$C_5H_{11}O_8P$	230.11	0.9	Carbohydrates	"14075-00-4"	530100	23670	849200	612000	34270	982000
36	beta-L-Arabinose	$C_5H_{10}O_5$	150.13	0.9	Carbohydrates	"5328-37-0"	64260	28850	73110	57240	45720	76640
37	Dihydroxyacetone phosphate	$C_3H_7O_6P$	170.058	0.9	Carbohydrates	"57-04-5"	199900	74810	34030	202500	116800	59530
38	D-Galactose	$C_6H_{12}O_6$	180.16	0.9	Carbohydrates	"59-23-4"	1370000	2762000	723900	1812000	2506000	1150000
39	Hydroxycitric acid	$C_6H_8O_8$	208.124	0.9	Organic Acids	"6205-14-7"	76070	36780	14200	67890	31880	20540
40	D-Xylonic acid	$C_5H_{10}O_6$	166.048	0.9	Organic Acids	"526-91-0"	3895000	4906000	491700	4288000	2499000	702300
41	Riboflavin-5'-monophosphate	$C_{17}H_{21}N_4O_9P$	456.344	0.91	Polyphenol	"146-17-8"	734900	21920	10840	1160000	17490	4023
42	Lactobionic acid	$C_{12}H_{22}O_{12}$	358.3	0.91	Carbohydrates	"96-82-2"	77770	8433	19413	139300	8276	57311
43	D-(+)-Cellobiose	$C_{12}H_{22}O_{11}$	342.3	0.91	Carbohydrates	"528-50-7"	63920	9040	7393	103400	53731	52582
44	D-Fructose-1,6-biphosphate	$C_6H_{14}O_{12}P_2$	339.996	0.91	Carbohydrates	"38099-82-0"	110900	67600	20710	119600	25480	21670
45	N-Acetylglucosamine 1-phosphate	$C_8H_{16}NO_9P$	301.1	0.91	Carbohydrates	"6866-69-9"	58490	91910	58113	116900	125100	115799
46	6-Phosphogluconic acid	$C_6H_{13}O_{10}P$	276.135	0.91	Carbohydrates	"921-62-0"	405100	119300	41780	569400	97020	43040
47	D-Gluconic acid	$C_6H_{12}O_7$	196.155	0.91	Carbohydrates	"526-95-4"	4663000	2059000	417900	6201000	2324000	450400
48	Lactose	$C_{12}H_{22}O_{11}$	342.3	0.911	Carbohydrates	"63-42-3"	169300	12550	5418	195600	12110	7614
49	alpha-D-Glucose	$C_6H_{12}O_6$	180.16	0.912	Carbohydrates	"492-62-6"	1262000	774300	172900	1265000	810500	364100

50	Fructose	$C_6H_{12}O_6$	180.16	0.92	Carbohydrates	"57-48-7"	502600	146100	47360	768900	160600	81680
51	D-Ribose	$C_5H_{10}O_5$	150.13	0.92	Carbohydrates	"50-69-1"	421500	709700	250900	439800	645500	560700
52	D-Galactonic acid	$C_6H_{12}O_7$	196.155	0.92	Organic Acids	"13382-27-9"	3066000	1395000	278500	3970000	1632000	299500
53	L-Arabinitol	$C_5H_{12}O_5$	152.15	0.925	Carbohydrates	"7643-75-6"	32090	7867	5975	52090	10280	18550
54	Ribitol	$C_5H_{12}O_5$	152.15	0.927	Carbohydrates	"488-81-3"	644800	128300	13840	805900	49310	29030
55	Ajugol	$C_{15}H_{24}O_9$	348.346	0.93	Terpenoids	"52949-83-4"	42750	14590	12990	21370	13360	24540
56	Oxalic acid	$C_2H_2O_4$	90.035	0.93	Organic Acids	"144-62-7"	2205000	215257	407900	2027000	397700	833900
57	D-Glucose	$C_6H_{12}O_6$	180.156	0.94	Carbohydrates	"50-99-7"	620500	1016000	218400	735700	915600	348800
58	L-Gulono-1,4-lactone	$C_6H_{10}O_6$	178.14	0.948	Carbohydrates	"1128-23-0"	424400	153700	124500	463300	181500	133900
59	Butein	$C_{15}H_{12}O_5$	272.069	0.95	Chalcones	"487-52-5"	85820	49890	12749	225100	31420	8595
60	Bisulfurous Acid	$C_{11}H_8O_2$	172.18	0.95	Quinones	"58-27-5"	308800	21410	16940	520700	45330	18030
61	L-Fucose	$C_6H_{12}O_5$	164.069	0.95	Carbohydrates	"2438-80-4"	213600	52200	52780	192200	64400	88090
62	D-3-Phosphoglyceric acid	$C_3H_5H_2O_7P$	230.021	0.95	Carbohydrates	"80731-10-8"	1408000	169400	80250	2131000	254300	81640
63	L-(+)-Rhamnose Monohydrate	$C_6H_{14}O_6$	182.17	0.96	Carbohydrates	"10030-85-0"	68060	16640	12390	97390	25730	56238
64	Trehalose 6-phosphate	$C_{12}H_{23}O_{14}P$	422.276	0.96	Carbohydrates	"4484-88-2"	172700	120100	52710	326000	121400	33040
65	Melibiose	$C_{12}H_{22}O_{11}$	342.3	0.96	Carbohydrates	"585-99-9"	429500	203700	34180	665800	53030	47040
66	D-Tagatose	$C_6H_{12}O_6$	180.156	0.96	Carbohydrates	"87-81-0"	1928000	4475000	1148000	2414000	3915000	1934000
67	alpha,alpha-Trehalose	$C_{12}H_{22}O_{11}$	342.296	0.962	Carbohydrates	"99-20-7"	90490	77810	14400	192000	43795	6896
68	Isomaltulose	$C_{12}H_{22}O_{11}$	342.116	0.97	Carbohydrates	"13718-94-0"	391000	244800	29180	800100	144396	43270
69	Coniferin	$C_{16}H_{22}O_8$	342.132	0.97	Carbohydrates	"531-29-3"	472800	255800	37430	1036000	86470	43040
70	3-Hydroxy-3-methyl butyric acid	$C_5H_{10}O_3$	118.063	0.97	Organic Acids	"625-08-1"	25160	288200	242300	34320	273900	296600
71	L-Rhamnose	$C_6H_{12}O_5$	164.16	0.98	Carbohydrates	"73-34-7"	115000	46700	21180	131700	42630	40520
72	Sucrose	$C_{12}H_{22}O_{11}$	342.116	0.98	Carbohydrates	"57-50-1"	106600	84630	14490	236700	29050	29650
73	Capsianoside I	$C_{32}H_{52}O_{14}$	660.336	0.98	Terpenoids	"121924-04-7"	907000	273700	252800	1290000	336200	265600
74	Angelol B	$C_{20}H_{24}O_7$	376.4	0.98	Coumarins	"83156-04-1"	27160	18880	7677	32960	11780	10040
75	Madecassic acid	$C_6H_{12}O_5$	164.156	0.99	Terpenoids	"18449-41-7"	23740	7635	7853	23780	12240	4783
76	Glycolic acid	$C_2H_4O_3$	76.05	0.99	Organic Acids	"79-14-1"	135900	291200	238500	154900	364000	461300
77	Quinic acid	$C_7H_{12}O_6$	192.17	0.998	Polyphenol	"77-95-2"	220100000	137600000	49360000	260200000	173300000	49220000
78	beta-D-Lactose	$C_{12}H_{22}O_{11}$	342.297	1	Carbohydrates	"5965-66-2"	139900	25810	17490	241100	43108	10470
79	Tartaric acid	$C_4H_6O_6$	150.09	1.008	Organic Acids	"87-69-4"	957100	394900	193800	1048000	768800	750000
80	Raffinose	$C_{18}H_{32}O_{16}$	504.437	1.009	Carbohydrates	"512-69-6"	28420	21160	7713	66070	10130	33363
81	Sakuranetin	$C_{16}H_{14}O_5$	286.279	1.01	Flavanones	"2957-21-3"	115300	52300	13200	157400	46080	9793
82	Maltose	$C_{12}H_{22}O_{11}$	342.3	1.01	Carbohydrates	"69-79-4"	82010	12980	7975	169600	52395	5470
83	D-Xylulose	$C_5H_{10}O_5$	150.13	1.01	Carbohydrates	"551-84-8"	25000	26710	16640	30530	25670	23700
84	D-Xylose	$C_5H_{10}O_5$	150.131	1.01	Carbohydrates	"58-86-6"	117900	267700	86020	126700	229200	169700
85	Cellotriose	$C_{18}H_{32}O_{16}$	504.437	1.02	Carbohydrates	"33404-34-1"	11200	26950	7694	12540	8367	5880
86	Gluconolactone	$C_6H_{10}O_6$	178.14	1.02	Carbohydrates	"90-80-2"	202600	96040	65400	289500	104100	83910
87	Kinetin 9-riboside	$C_{15}H_{17}N_5O_5$	347.326	1.03	Anthocyanins	"4338-47-0"	9230	10450	242200	15090	18080	201500
88	Glyceraldehyde 3-phosphate	$C_3H_7O_6P$	170.058	1.03	Carbohydrates	"142-10-9"	6735	43130	33170	6016	187200	59500
89	Guanidinoethyl Sulfonate	$C_3H_9N_3O_3S$	167.187	1.03	Organic Acids	"543-18-0"	12230	12401	7374	20150	10869	13360
90	Shikimic Acid	$C_7H_{10}O_5$	174.15	1.038	Polyphenol	"138-59-0"	33840000	33690000	5797000	36240000	33750000	4238000
91	P-Coumaryl Alcohol	$C_9H_{10}O_2$	150.174	1.04	Phenylpropanoids	"3690-05-9"	88670	338300	163300	114400	261900	255100
92	D-Erythrose 4-phosphate	$C_4H_9O_7P$	200.084	1.08	Carbohydrates	"585-18-2"	151500	68510	63640	249000	199300	59190
93	L(-)-Malic acid	$C_4H_6O_5$	134.022	1.08	Organic Acids	"636-61-3"	103700000	68820000	40290000	117000000	151900000	95580000
94	Sucralose	$C_{12}H_{19}Cl_3O_8$	397.634	1.1	Carbohydrates	"56038-13-2"	21100	8414	9628	12830	6135	11400
95	Fumaric Acid	$C_4H_4O_4$	116.072	1.11	Organic Acids	"110-17-8"	3431000	2424000	1388000	4067000	4966000	3548000
96	8-Gingerol	$C_{43}H_{32}O_{20}$	868.702	1.13	Polyphenol	"30462-35-2"	39740	27100	19560	36930	50960	29840
97	3-Hydroxybutyrate	$C_4H_8O_3$	104.104	1.13	Organic Acids	"300-85-6"	2210000	2081000	1501000	2770000	5432000	5210000
98	Malonic acid	$C_3H_4O_4$	104.011	1.19	Organic Acids	"141-82-2"	4126000	4031000	3003000	5194000	10200000	10200000

99	3-Hydroxypropanoic acid	$C_3H_6O_3$	90.032	1.21	Organic Acids	"503-66-2"	165000	327900	414500	188700	462800	983300
100	Chrysin	$C_{15}H_{10}O_4$	254.24	1.284	Flavones	"480-40-0"	80210	7014	19336	137200	5815	56441
101	D-threo-Isocitric acid	$C_6H_8O_7$	192.124	1.41	Organic Acids	"6061-97-8"	41770000	17660000	11620000	38000000	53710000	35790000
102	D-Lactic acid	$C_3H_6O_3$	90.08	1.44	Organic Acids	"10326-41-7"	84380	1855000	658000	52030	2804000	1029000
103	L-Citramalic acid	$C_5H_8O_5$	148.037	1.46	Organic Acids	"6236-09-5"	1865000	661400	492900	2193000	1520000	1259000
104	Carvacrol	$C_{10}H_{14}O$	150.22	1.467	Terpenoids	"499-75-2"	8944	20280	46200	32565	20120	23970
105	D-Arabinono-1,4-lactone	$C_5H_8O_5$	148.037	1.48	Carbohydrates	"2782-09-4"	1323000	1201000	357400	1933000	1722000	1066000
106	Lactic acid	$C_3H_6O_3$	90.078	1.48	Organic Acids	"50-21-5"	1216000	25550000	9295000	711900	39610000	14730000
107	L-Lactic acid	$C_3H_6O_3$	90.08	1.487	Organic Acids	"79-33-4"	63670	1322000	427200	36370	2170000	739600
108	2–furanoic acid	$C_5H_4O_3$	112.084	1.49	Organic Acids	"88-14-2"	13310000	5873000	3772000	15000000	12800000	10910000
109	Isocitrate	$C_6H_8O_7$	192.124	1.49	Organic Acids	"320-77-4"	162000000	68910000	48470000	198600000	164400000	134100000
110	Citric acid	$C_6H_8O_7$	192.12	1.516	Organic Acids	"77-92-9"	119400000	53440000	34320000	143300000	122200000	88850000
111	D-Galacturonic acid	$C_6H_{10}O_7$	194.139	1.56	Carbohydrates	"685-73-4"	3336000	1470000	1175000	3752000	3259000	2836000
112	cis-Aconitic acid	$C_6H_6O_6$	174.11	1.766	Organic Acids	"585-84-2"	687400	3088000	225400	961800	2396000	3519000
113	Acetoxyacetic acid	$C_4H_6O_4$	118.027	1.89	Organic Acids	"13831-30-6"	7994	9545	6081	6769	28980	23360
114	Succinic acid	$C_4H_6O_4$	118.09	1.936	Organic Acids	"110-15-6"	25550000	23860000	28640000	28360000	54380000	73860000
115	Methylmalonate	$C_4H_6O_4$	118.09	1.938	Organic Acids	"516-05-2"	9117000	8341000	9609000	10960000	21520000	28760000
116	Citramalate	$C_5H_8O_5$	148	2.04	Organic Acids	"2306-22-1"	390300	481100	369000	508700	1395000	1534000
117	Monotropein	$C_{16}H_{22}O_{11}$	390.34	2.2	Terpenoids	"5945-50-6"	8552	4357	4059	15650	15600	29320
118	cis-4-Hydroxy-D-proline	$C_5H_9NO_3$	131.13	2.26	Organic Acids	"2584-71-6"	102500	59190	6516	153300	64560	8547
119	1,6-Anhydro-beta-D-Glucopyranose	$C_6H_{10}O_5$	162.141	2.3	Carbohydrates	"498-07-7"	702200	598300	627600	874600	1935000	2286000
120	Arbutin	$C_{12}H_{16}O_7$	272.25	2.32	Carbohydrates	"497-76-7"	108800	277500	456300	114200	428000	951500
121	alpha-Arbutin	$C_{12}H_{16}O_7$	272.25	2.33	Carbohydrates	"84380-01-8"	355900	988300	1719000	385900	1557000	3631000
122	beta-Hydroxyisobutyrate	$C_4H_8O_3$	104.104	2.4	Organic Acids	"2068-83-9"	35890	112600	52690	34510	182100	122000
123	Dl-3,4-Dihydroxymandelic acid	$C_8H_8O_5$	184.037	2.41	Polyphenol	"775-01-9"	293800	488700	475700	328100	933900	1122000
124	10-Deacetylasperulosidic acid	$C_{16}H_{22}O_{11}$	390.344	2.56	Terpenoids	"14259-55-3"	26290	21420	26040	27870	51780	75150
125	2-Methyllactic acid	$C_4H_8O_3$	104.1	2.597	Organic Acids	"594-61-6"	125800	235500	208300	158100	534200	522000
126	Laricitrin	$C_{16}H_{12}O_8$	332.262	2.6	Flavonols	"53472-37-0"	3083000	134200	116500	2736000	775800	39970
127	trans-2,3,4-Trimethoxycinnamic Acid	$C_{12}H_{14}O_5$	238.237	2.65	Phenylpropanoids	"33130-03-9"	50717	16380	27180	50126	35010	67970
128	2-Hydroxybutyric acid	$C_4H_8O_3$	104.1	2.741	Organic Acids	"600-15-7"	125900	1184000	874600	149300	1959000	633200
129	Dl-Glyceraldehyde 3-phosphate	$C_3H_7O_6P$	169.998	2.76	Carbohydrates	"591-59-3"	50500000	279800000	347200000	57480000	406300000	553600000
130	Pyrogallol	$C_6H_6O_3$	126.11	2.777	Polyphenol	"87-66-1"	18990000	117700000	138300000	22290000	158300000	233000000
131	Gallic acid	$C_7H_6O_5$	170.12	2.78	Polyphenol	"149-91-7"	154000000	772900000	900100000	178000000	1020000000	1291000000
132	Levulinic acid	$C_5H_8O_3$	116.12	3.489	Organic Acids	"123-76-2"	71270	33110	75250	129300	80310	242500
133	Guaiacol	$C_7H_8O_2$	124.137	3.62	Polyphenol	"90-05-1"	752800	367500	1851000	1008000	876000	10530000
134	Kinsenoside	$C_{10}H_{16}O_8$	264.229	3.93	Carbohydrates	"151870-74-5"	19770	6279	12942	24210	9414	23099
135	Homogentisic Acid	$C_8H_8O_4$	168.15	4.059	Organic Acids	"451-13-8"	76370	217126	271025	133000	240600	422300
136	Diethyl phosphate	$C_4H_{11}O_4P$	154	4.2	Organic Acids	"598-02-7"	5615	16850	39330	3561	17880	180800
137	Androsin	$C_{15}H_{20}O_8$	328.319	4.3	Polyphenol	"531-28-2"	96190	236100	36920	96560	72120	55730
138	3,5-Dihydroxybenzoic acid	$C_7H_6O_4$	154.12	4.461	Benzoic acids	"99-10-5"	122100000	26400000	76960000	133900000	42100000	144000000
139	Danshensu	$C_9H_{10}O_5$	198.17	4.473	Polyphenol	"76822-21-4"	16240	2552000	9447000	23120	7127000	17250000
140	(-)-Gallocatechin	$C_{15}H_{14}O_7$	306.267	4.51	Flavonoids	"3371-27-5"	32140	3639	36260	37520	57360	160800
141	Catalpol	$C_{15}H_{22}O_{10}$	362.333	4.55	Terpenoids	"2415-24-9"	216300	72770	313500	195200	234783	498100
142	1-O-Gentisoyl-D-glucoside	$C_{13}H_{16}O_9$	316.079	4.56	Polyphenol	"23445-11-6"	1601000	1668000	1415000	1823000	2783000	2483000
143	3-Aminosalicylic Acid	$C_7H_7NO_3$	153.135	4.57	Organic Acids	"570-23-0"	227700	483100	241700	238400	266500	571600
144	Protocatechuic acid	$C_7H_6O_4$	154.12	4.571	Polyphenol	"99-50-3"	221400000	50380000	134800000	242100000	74730000	245300000
145	Geniposidic acid	$C_{16}H_{22}O_{10}$	374.34	4.615	Terpenoids	"27741-01-1"	111500	31580	18060	76235	24080	65270
146	2,4-Dihydroxybenzoic acid	$C_7H_6O_4$	154.12	4.623	Organic Acids	"89-86-1"	18080000	3615000	10430000	20810000	5692000	21620000
147	1-O-Galloyl-D-glucose	$C_{13}H_{16}O_{10}$	332.074	4.68	Polyphenol	"58511-73-2"	50290	620000	145700	154553	428200	421744

148	Koaburaside	$C_{14}H_{20}O_9$	332.111	4.68	Polyphenol	"41653-73-0"	22750	1263000	227100	24510	614000	257000
149	Methyl salicylate-2-O-glucoside	$C_{14}H_{18}O_8$	314.1	4.8	Polyphenol	"10019-60-0"	193645	42930	237100	278655	68870	508500
150	3-Hydroxy-glabrol	$C_{25}H_{28}O_5$	40.499	4.82	Flavonoids	"74148-41-7"	110458	37780	12830	133062	96090	99310
151	Neochlorogenic acid	$C_{16}H_{18}O_9$	354.309	4.879	Polyphenol	"906-33-2"	20620	9170	12763	28810	15396	30100
152	Mandelic acid	$C_8H_8O_3$	152.047	4.91	Organic Acids	"17199-29-0"	340100	542800	255700	371000	489400	503500
153	2-Methylpentanedioic acid	$C_6H_{10}O_4$	146.14	5.012	Organic Acids	"617-62-9"	49100	227700	208700	51860	367900	563700
154	Esculin	$C_{15}H_{16}O9$	340.28	5.145	Coumarins	"531-75-9"	145700	55880	27060	181100	90770	66160
155	Procyanidin B3	$C_{30}H_{26}O_{12}$	578.142	5.15	Anthocyanins	"23567-23-9"	580451	130400	235800	977694	1051000	48550
156	Procyanidin B2	$C_{30}H_{26}O_{12}$	578.529	5.16	Anthocyanins	"29106-49-8"	600913	180200	303700	955008	1117000	81750
157	Bergenin	$C_{14}H_{16}O_9$	328.27	5.196	Phenylpropanoids	"477-90-7"	11366	12590	9945	14468	11987	17290
158	Cyanidin 3-O-glucoside	$C_{21}H_{21}ClO_{11}$	484.84	5.21	Anthocyanins	"7084-24-4"	606000	488000	243100	760200	810400	425300
159	Idaein chloride	$C_{21}H_{21}ClO_{11}$	484.84	5.213	Anthocyanins	"27661-36-5"	74562	49080	35173	80016	113800	62300
160	Petunidin-3-O-glucoside chloride	$C_{22}H_{23}O_{12}Cl$	514.865	5.282	Anthocyanins	"6988-81-4"	8488	3741	22380	5056	41830	43090
161	4-Hydroxy-3-methoxycinnamic acid	$C_{10}H_{10}O_4$	194.0579	5.34	Phenylpropanoids	"537-98-4"	97690	112500	85170	110156	151800	176500
162	Sieboldin	$C_{21}H_{24}O_{11}$	452.131	5.35	Flavonoids	"18777-73-6"	39830	25780	26203	32160	49285	64230
163	(+)-Catechin	$C_{15}H_{14}O_6$	290.27	5.37	flavanol	"154-23-4"	121526	27690	15300	150911	129700	45681
164	Ophiopogonanone C	$C_{19}H_{16}O_7$	356.332	5.38	Flavonoids	"477336-75-7"	11940	104400	66230	15840	146800	106300
165	2,5-Dihydroxybenzoic acid	$C_7H_6O_4$	154.12	5.44	Benzoic acids	"490-79-9"	3205000	137800	1818000	4340000	510700	5509000
166	Cryptochlorogenic acid	$C_{16}H_{18}O_9$	354.309	5.443	Polyphenol	"905-99-7"	197700	34281	32272	199900	66498	67180
167	1-Caffeoylquinic acid	$C_{16}H_{18}O_9$	354.095	5.46	Polyphenol	"1241-87-8"	3669000	508200	183900	3598000	532300	5893000
168	2-(Formylamino)benzoic acid	$C_8H_7NO_3$	165.043	5.47	Benzoic acids	"3342-77-6"	26900	18925	10977	29620	28311	28640
169	(2S)-2-Isopropylmalate	$C_7H_{12}O_5$	176.068	5.52	Organic Acids	"49601-06-1"	459000	37920	190100	514300	134500	656100
170	Methyl gallate	$C_8H_8O_5$	100.12	5.523	Benzoic acids	"99-24-1"	14270000	35370000	16070000	14860000	100900000	45860000
171	3-O-Feruloylquinic acid	$C_{17}H_{20}O_9$	368.335	5.55	Polyphenol	"1899-29-2"	22790	48080	32051	46050	157900	20170
172	Warfarin Sodium	$C_{19}H_{15}O_4$	330.31	5.56	Coumarins	"129-06-6"	74710	70070	27810	128500	75880	136500
173	Swertiamarin	$C_{16}H_{22}O_{10}$	374.34	5.578	Terpenoids	"17388-39-5"	8409	71370	37100	39672	239500	31470
174	(R)-Mandelic acid	$C_8H_8O_3$	152.147	5.661	Organic Acids	"611-71-2"	584968	121100	304900	907743	184400	1443000
175	Corilagin	$C_{27}H_{22}O_{18}$	634.453	5.7	Polyphenol	"23094-69-1"	17540000	2033380	1526000	21610000	4881000	4721000
176	Dihydrokaempferol	$C_{15}H_{12}O_6$	288.063	5.75	Flavonoids	"480-20-6"	243100	66520	81073	298400	155500	162900
177	Acetylharpagide	$C_{17}H_{26}O_{11}$	406.38	5.783	Terpenoids	"6926-14-3"	20410	44220	23870	3610	273100	51530
178	Caffeic acid	$C_9H_8O_4$	180.16	5.801	Phenylpropanoids	"331-39-5"	169400	5120000	21750000	113300	7394000	31140000
179	Terephthalic acid	$C_8H_6O_4$	166.13	5.801	Benzoic acids	"100-21-0"	484000	32790000	28560000	401400	37720000	49120000
180	Esculetin	$C_9H_6O_4$	178.14	5.832	Coumarins	"305-01-1"	12210	13540	16470	18280	6959	20640
181	Protosappanin B	$C_{16}H_{16}O_6$	304.295	5.86	Polyphenol	"102036-29-3"	137130	250300	219800	55070	251500	440600
182	Epigallocatechin 3-gallate	$C_{22}H_{18}O_{11}$	458.37	5.874	Polyphenol	"989-51-5"	41950	54096	20110	125800	190400	62160
183	1-O-b-D-glucopyranosyl sinapate	$C_{17}H_{22}O_{10}$	386.351	5.88	Polyphenol	"78185-48-5"	92770	163600	39630	118300	176900	134700
184	(-)-Epicatechin	$C_{15}H_{14}O_6$	290.27	5.9	flavanol	"490-46-0"	1961000	1470000	1270000	2204000	2788000	1384000
185	Vanillic acid	$C_8H_8O_4$	168.15	5.901	Organic Acids	"121-34-6"	674000	424300	1839000	764700	792200	4385000
186	3-O-p-Coumaroylquinic acid	$C_{16}H_{18}O_8$	338.1	5.92	Polyphenol	"87099-71-6"	32500000	278100	764400	28720000	720300	279500
187	4-O-p-Coumaroylquinic acid	$C_{16}H_{18}O_8$	338.1	5.93	Polyphenol	"32451-86-8"	4349000	36360	105400	4908000	88630	33040
188	L-3-Phenyllactic Acid	$C_9H_{10}O_3$	166.174	6.03	Phenylpropanoids	"20312-36-1"	41160	128700	78648	53940	193200	134803
189	Isoschaftoside	$C_{26}H_{28}O_{14}$	564.499	6.03	Flavones	"52012-29-0"	925000	705300	211900	1070000	798200	261300
190	Homovanillic acid	$C_9H_{10}O_4$	182.17	6.091	Organic Acids	"306-08-1"	35350	217205	453800	88750	241600	809900
191	Isovanillic acid	$C_8H_8O_4$	168.15	6.1	Benzoic acids	"645-08-9"	1197000	977200	3896000	1548000	1613000	10220000
192	2,6-Dihydroxybenzoic acid	$C_7H_6O_4$	154.12	6.13	Benzoic acids	"303-07-1"	1163000	685000	4824000	1460000	1212000	5984000
193	Quercetin 3-O-sophoroside	$C_{27}H_{30}O_{17}$	626.524	6.13	Alkaloids	"18609-17-1"	128800	364400	152800	144400	339900	266500
194	2-Isopropylmalic acid	$C_7H_{12}O_5$	176.167	6.13	Organic Acids	"3237-44-3"	13721	11265	12490	15500	4420	12060
195	Isoorientin	$C_{21}H_{20}O_{11}$	448.38	6.174	Flavones	"4261-42-1"	161400	45300	25097	174600	309200	23660
196	2,3-Dihydroxybenzoic acid	$C_7H_6O_4$	154.027	6.21	Benzoic acids	"303-38-8"	281600	238600	1657000	472300	418300	2416000

197	Secoisolariciresinol diglucoside	$C_{32}H_{46}O_{16}$	686.7	6.224	Lignans	"148244-82-0"	15080	21640	8973	9563	14330	7775
198	E-3,4,5'-Trihydroxy-3'-glucopyranosylstilbene	$C_{20}H_{22}O_9$	406.4	6.23	Phenylpropanoids	"29884-49-9"	62280	59440	54610	63983	84660	85360
199	Glucosylvitexin	$C_{27}H_{30}O_{15}$	594.526	6.25	Flavonoids	"76135-82-5"	4127000	4753000	1320000	4417000	5085000	1668000
200	Cyanidin chloride	$C_{15}H_{11}ClO_6$	322.7	6.263	Anthocyanins	"528-58-5"	57610	9719	29910	90890	107200	95613
201	Saponarin	$C_{27}H_{30}O_{15}$	594.518	6.27	Flavonoids	"20310-89-8"	535700	589500	109700	613600	734900	170600
202	Plantamajoside	$C_{29}H_{36}O_{16}$	640.587	6.35	Phenylpropanoids	"104777-68-6"	18810	6822	3462	17600	14310	20633
203	Rutin hydrate	$C_{27}H_{30}O_{16}.xH_2O$	610.52	6.36	Flavonoids	"207671-50-9"	16790000	34600000	26830000	16620000	69000000	35740000
204	Isohemiphloin	$C_{21}H_{22}O10$	434.121	6.39	Flavanones	"3682-02-8"	241200	95860	207900	234900	89990	119100
205	2-Hydroxyisocaproic Acid	$C_6H_{12}O_3$	132.158	6.39	Organic Acids	"498-36-2"	15150	31560	81490	14040	62150	215800
206	Vitexin-2-O-rhaMnoside	$C_{27}H_{30}O_{14}$	578.52	6.411	Flavonoids	"64820-99-1"	292200	345200	103500	302700	526900	157500
207	Spinosin	$C_{28}H_{32}O_{15}$	608.545	6.418	Flavones	"72063-39-9"	142462	249100	123940	168375	261300	190439
208	Neohesperidin dihydrochalcone	$C_{28}H_{36}O_{15}$	612.576	6.43	Flavanones	"20702-77-6"	235300	513400	311300	222900	877800	467700
209	2-Hydroxycinnamate	$C_9H_8O_3$	164.047	6.48	Phenylpropanoids	"583-17-5"	2496000	32180000	250300000	2403000	47890000	326700000
210	Rutin	$C_{27}H_{30}O_{16}$	610.518	6.51	Flavones	"153-18-4"	42610000	29430000	68460000	37000000	58040000	130600000
211	(-)-Catechin Gallate	$C_{22}H_{18}O_{10}$	442.379	6.51	flavanol	"130405-40-2"	141400	438600	42400	117900	1838000	21220
212	Isomucronulatol-7-O-glucoside	$C_{23}H_{28}O_{10}$	464.469	6.53	Flavonoids	"94367-43-8"	2058000	1769000	12660000	2025000	4093000	20490000
213	p-Hydroxy-cinnamic acid	$C_9H_8O_3$	164.16	6.531	Phenylpropanoids	"7400-08-0"	2234000	21380000	176900000	2245000	30000000	241200000
214	Isovitexin	$C_{21}H_{20}O_{10}$	432.378	6.533	Flavones	"29702-25-8"	1930000	169800	175200	2567000	241300	150900
215	Rosmarinic acid-3'-O-glucoside	$C_{24}H_{26}O_{13}$	522.137	6.54	Polyphenol	"910028-78-3"	260300	1872000	485000	294000	1425000	952300
216	Ellagic acid	$C_{14}H_6O_8$	302.19	6.54	Polyphenol	"476-66-4"	23230000	49960000	39540000	22510000	89740000	51590000
217	Epicatechin Gallate	$C_{22}H_{18}O_{10}$	442.379	6.54	Flavonoids	"1257-08-5"	123700	361300	46490	103900	1332000	19880
218	Myricitrin	$C_{21}H_{20}O_{12}$	464.38	6.547	Flavonols	"17912-87-7"	2062000	1987000	14950000	1934000	4458000	22510000
219	p-Coumaric Acid	$C_9H_8O_3$	164.16	6.549	Phenylpropanoids	"501-98-4"	2347000	25940000	178000000	2403000	33690000	210800000
220	Kaempferol 3-O-robinobioside	$C_{27}H_{30}O_{15}$	594.159	6.55	Flavonols	"17297-56-2"	11580000	11180000	23700000	11810000	27030000	47070000
221	Hyperoside	$C_{21}H_{20}O_{12}$	464.38	6.554	Flavonols	"482-36-0"	1749000	1521000	11720000	1801000	3248000	18190000
222	Vitexin	$C_{21}H_{20}O_{10}$	432.38	6.559	Flavones	"3681-93-4"	1704000	152700	178600	2232000	231000	181000
223	Icariside E5	$C_{26}H_{34}O_{11}$	522.21	6.56	Phenylpropanoids	"126176-79-2"	170100	1128000	507300	213500	1362000	701300
224	Lariciresinol-4'-O-glucoside	$C_{26}H_{34}O_{11}$	522.211	6.56	Lignans	"143663-00-7"	213500	1208000	420000	278300	1311000	630100
225	Tricin O-saccharic acid	$C_{23}H_{22}O_{14}$	522.1	6.58	Flavonoids		1204000	8197000	2834000	1336000	8167000	3972000
226	Rehmannioside D	$C_{27}H_{42}O_{20}$	686.6177	6.58	Terpenoids	"81720-08-3"	21550	2432	5440	50190	11500	19802
227	Scutellarin	$C_{21}H_{18}O_{12}$	462.36	6.59	Flavonoids	"27740-01-8"	67480	6168	85360	50390	23750	108400
228	Isotrifoliin	$C_{21}H_{20}O_{12}$	464.096	6.59	Flavonoids	"21637-25-2"	3324000	2778000	20740000	2722000	6343000	32430000
229	Spiraeoside	$C_{21}H_{20}O_{12}$	464.096	6.59	Flavonoids	"20229-56-5"	3310000	3105000	22160000	3138000	6637000	34230000
230	Isoquercitrin	$C_{21}H_{20}O_{12}$	464.38	6.596	Flavonols	"482-35-9"	4085000	4378000	29170000	3704000	9518000	45500000
231	Ethyl gallate	$C_9H_{10}O_5$	198.17	6.611	Benzoic acids	"831-61-8"	291400	9340000	207700	340700	23550000	456900
232	Trifolin	$C_{21}H_{20}O_{11}$	448.101	6.62	Flavonoids	"23627-87-4"	979100	1131000	54980000	1150000	1430000	67890000
233	1-Hydroxypinoresinol-1-O-Glucoside	$C_{26}H_{32}O_{12}$	536.189	6.62	Lignans	"81495-71-8"	32780	51980	23610	35260	128100	47340
234	Kaempferitrin	$C_{27}H_{30}O_{14}$	578.519	6.641	Flavonols	"482-38-2"	35137	105609	215900	6981	127880	359800
235	Octanedioic acid	$C_8H_{14}O_4$	174.19	6.649	Organic Acids	"505-48-6"	708500	515200	6398000	879100	780000	18480000
236	Kaempferol7-O-beta-D-glucopyranoside	$C_{21}H_{20}O_{11}$	448.377	6.65	Flavonols	"16290-07-6"	775400	597300	61670000	853700	823000	69220000
237	Luteolin 7-O-glucoside	$C_{21}H_{20}O_{11}$	448.38	6.664	Flavonoids	"5373-11-5"	1103000	1307000	60150000	1155000	1821000	72220000
238	Apigenin 5-O-glucoside	$C_{21}H_{20}O_{10}$	432.38	6.68	Flavonoids	"28757-27-9"	60060	18538	26350	98040	40470	47700
239	Kaji-ichigoside F1	$C_{36}H_{58}O_{10}$	650.85	6.71	Terpenoids	"95298-47-8"	454500	16800	220600	419500	63080	91660
240	Lonicerin	$C_{27}H_{30}O_{15}$	594.526	6.73	Flavonoids	"20633-84-5"	14130000	11480000	26080000	12810000	28070000	47600000
241	Hydroferulic acid	$C_{10}H_{12}O_4$	196.2	6.74	Phenylpropanoids	"1135-23-5"	972400	272000	468700	1764000	561700	297928
242	Astilbin	$C_{21}H_{22}O_{11}$	450.39	6.745	Flavonoids	"29838-67-3"	88860	15380	235400	71260	46260	179400
243	Wikstromol	$C_{20}H_{22}O_7$	374.39	6.78	Lignans	"61521-74-2"	119181	27928	57080	163085	23930	105700
244	Sesamol	$C_7H_6O_3$	138.12	6.79	Polyphenol	"533-31-3"	9482	4241	35260	7511	17267	54690
245	Isorhamnetin-3-O-neohespeidoside	$C_{28}H_{32}O_{16}$	624.552	6.79	Flavonoids	"55033-90-4"	69610	51460	76050	63150	157100	104200

246	3-Phenyllactic acid	$C_9H_{10}O_3$	166.174	6.81	Organic Acids	"828-01-3"	991100	60500	243400	1395000	154900	753900
247	Narirutin	$C_{27}H_{32}O_{14}$	580.535	6.837	Flavanones	"14259-46-2"	119300	38480	10380	42060	60500	68138
248	Tectoridin	$C_{22}H_{22}O_{11}$	462.4	6.864	Isoflavonoids	"611-40-5"	162300	44440	136800	173700	117900	195200
249	Antiarol	$C_9H_{12}O_4$	184.19	6.88	Polyphenol	"642-71-7"	216100	7677	47382	245800	9756	100457
250	Ferulic Acid	$C_{10}H_{10}O_4$	194.19	6.881	Polyphenol	"1135-24-6"	444500	3451000	27500000	517000	3084000	50170000
251	Taxifolin	$C_{15}H_{12}O_7$	304.25	6.885	Flavonoids	"480-18-2"	393100	363500	415200	554600	1260000	653800
252	Pelargonidin chloride	$C_{15}H_{11}ClO_5$	306.7	6.91	Anthocyanins	"134-04-3"	19350	121100	121400	124454	889600	24990
253	Pheophorbide A	$C_{35}H_{36}N_4O_5$	592.684	6.93	Organic Acids	"15664-29-6"	35770	12960	24080	39630	11660	25940
254	Rhapontin	$C_{21}H_{24}O_9$	420.41	6.94	Phenylpropanoids	"155-58-8"	105550	73600	46700	121487	73280	115000
255	Lirioresinol A	$C_{22}H_{26}O_8$	418.162	6.96	Lignans	"21453-71-4"	20640	292300	63650	30880	82660	96970
256	Methyl protocatechuate	$C_8H_8O_4$	168.15	6.97	Benzoic acids	"2150-43-8"	1020000	2064000	687900	1531000	3701000	1925000
257	Vincetoxicoside B	$C_{21}H_{20}O_{11}$	448.383	6.97	Flavonoids	"22007-72-3"	1271000	375100	4226000	962700	792000	3171000
258	Naringin	$C_{27}H_{32}O_{14}$	580.53	6.979	Flavanones	"10236-47-2"	101400	35920	29000	61270	33320	61860
259	3-Hydroxycinnamic acid	$C_9H_8O_3$	164.16	6.98	Phenylpropanoids	"14755-02-3"	5011710	3829000	25750000	5258400	12403800	67800000
260	Liquiritin	$C_{21}H_{22}O_9$	418.39	6.98	Flavanones	"551-15-5"	164877	13710	123600	207717	14070	247100
261	Piceatannol	$C_{14}H_{12}O_4$	244.24	6.987	Phenylpropanoids	"10083-24-6"	57517	44580	56540	79769	120300	174200
262	Coniferyl Alcohol	$C_{10}H_{12}O_3$	180.2	7.05	Polyphenol	"458-35-5"	41910	34510	70070	41550	86450	173600
263	Pinobanksin	$C_{15}H_{12}O_5$	272.069	7.06	Flavonoids	"548-82-3"	387000	16910	146200	503200	58690	139100
264	Kaempferol 3-A-L-Arabinopyranoside	$C_{20}H_{18}O_{10}$	418.351	7.08	Flavonoids	"99882-10-7"	46390	42790	844000	22870	66490	1216000
265	Engeletin	$C_{21}H_{22}O10$	434.393	7.149	Flavonoids	"572-31-6"	251100	18740	62850	396500	62170	47820
266	Theaflavin	$C_{29}H_{24}O_{12}$	564.502	7.16	Flavonoids	"4670-05-7"	42258	34260	34942	11360	18740	64240
267	(+)-Isolariciresinol	$C_{20}H_{24}O_6$	360.407	7.21	Lignans	"548-29-8"	22660	14206	31170	63470	6717	23340
268	Pinoresinol-glucoside	$C_{26}H_{32}O_{11}$	520.526	7.23	Lignans	"69251-96-3"	135403	47420	159900	162099	63540	227600
269	Quercitrin	$C_{21}H_{20}O_{11}$	448.38	7.24	Flavonols	"522-12-3"	2899000	792400	9078000	2602000	1549000	6855000
270	Phlorizin	$C_{21}H_{24}O_{10}$	436.41	7.24	Flavonoids	"60-81-1"	33094	118700	88740	25750	172200	64190
271	Coniferylaldehyde	$C_{10}H_{10}O_3$	178.063	7.29	Polyphenol	"20649-42-7"	325200	51840	478700	442000	35520	2050000
272	alpha-Zearalanol	$C_{18}H_{26}O_5$	322.396	7.3	Phenylpropanoids	"26538-44-3"	296500	333417	343300	307300	627100	511800
273	Azaleatin	$C_{16}H_{12}O_7$	316.265	7.3	Flavonols	"529-51-1"	1839000	122800	934800	1805000	238800	1941000
274	Ayapin	$C_{11}H_8O_3$	188.041	7.3	Coumarins	"494-56-4"	2112000	743500	12310000	2788000	1323000	28840000
275	Eucommiol	$C_9H_{16}O_4$	188.105	7.31	Terpenoids	"55930-44-4"	8314000	3603000	36460000	10450000	5925000	70930000
276	Gossypetin	$C_{15}H_{10}O_8$	318.24	7.34	Flavonols	"489-35-0"	409500	41580	96270	437200	57650	95290
277	Myricetin	$C_{15}H_{10}O_8$	318.24	7.34	Flavonols	"529-44-2"	553600	71410	207400	647800	142100	253500
278	Kaempferin	$C_{21}H_{20}O_{10}$	432.106	7.35	Flavonoids	"482-39-3"	473700	88210	539900	389300	262700	471200
279	Kaempferol 7-O-rhamnoside	$C_{21}H_{20}O_{10}$	432.106	7.36	Flavonols	"20196-89-8"	567300	100500	722000	462700	321700	582000
280	Matairesinoside	$C_{26}H_{32}O_{11}$	520.533	7.38	Lignans	"23202-85-9"	8651	30760	69730	21510	98910	217500
281	Ginsenoside Re	$C_{48}H_{82}O_{18}$	947.15	7.43	Terpenoids	"52286-59-6"	13481	5395	7385	14510	5866	27390
282	8-O-Methylretusin	$C_{17}H_{14}O_5$	298.295	7.49	Isoflavonoids	"37816-20-9"	5332	11043	5637	3942	13452	40030
283	Trilobatin	$C_{21}H_{24}O_{10}$	436.409	7.523	Dihydrochalcones	"4192-90-9"	99715	80560	63730	96491	122300	50970
284	Methyl-Hesperidin	$C_{29}H_{38}O_{16}$	642.59	7.569	Flavonoids	"11013-97-1"	262200	58914	67416	260900	82168	174383
285	Baicalin	$C_{21}H_{18}O_{11}$	446.36	7.575	Flavones	"21967-41-9"	20420	8067	8598	22370	7487	8608
286	Veratric acid	$C_9H_{10}O_4$	182.17	7.65	Benzoic acids	"93-07-2"	6137	2573	6641	16850	15620	5827
287	Isovanillin	$C_8H_8O_3$	152.15	7.676	Polyphenol	"621-59-0"	66560	65420	48350	65860	725500	87010
288	Tiliroside	$C_{30}H_{26}O_{13}$	594.52	7.879	Flavonols	"20316-62-5"	14950000	233200	321900	15320000	163600	135300
289	Trifolirhizin	$C_{22}H_{22}O_{10}$	446.404	7.98	Isoflavonoids	"6807-83-6"	2309	8029	6528	5576	24160	4483
290	Eriodictyol	$C_{15}H_{12}O_6$	288.252	7.999	Flavanones	"552-58-9"	639800	38480	41370	760200	49850	49160
291	2'-Hydroxygenistein	$C_{15}H_{12}O_6$	286.048	8.02	Isoflavonoids	"1156-78-1"	58570	23546	29167	86530	47538	96310
292	Poncirin	$C_{28}H_{34}O_{14}$	594.561	8.049	Flavanones	"14941-08-3"	8054000	337500	251500	8353000	135400	67340
293	Morin	$C_{15}H_{10}O_7$	302.043	8.05	Flavonoids	"6472-38-4"	203500000	6062000	34590000	207700000	42810000	45990000
294	Luteolin	$C_{15}H_{10}O_6$	286.24	8.06	Flavones	"491-70-3"	182500	24670	25920	260000	40370	275800

295	Aleuritic Acid	$C_{16}H_{32}O_5$	304.42	8.09	Organic Acids	"533-87-9"	260500	6989	602400	364800	26990	1442000
296	Harpagoside	$C_{24}H_{30}O_{11}$	494.488	8.099	Terpenoids	"19210-12-9"	271100	60450	163000	274900	251200	291900
297	Sebacic acid	$C_{10}H_{18}O_4$	202.248	8.111	Organic Acids	"111-20-6"	2472000	1473000	12000000	3111000	2709000	32190000
298	Wedelolactone	$C_{16}H_{10}O_7$	314.246	8.229	Flavonoids	"524-12-9"	127907	17170	30830	106731	69020	40717
299	Wogonoside	$C_{22}H_{20}O_{11}$	460.388	8.26	Flavonoids	"51059-44-0"	2841	9384	5150	7740	18570	15627
300	Ethyl caffeate	$C_{11}H_{12}O_4$	208.211	8.507	Phenylpropanoids	"102-37-4"	10792	2717	2034	3166	54230	2985
301	trans-Cinnamic acid	$C_9H_8O_2$	148.16	8.507	Phenylpropanoids	"140-10-3"	8504	7054	4195	9573	7890	5668
302	Naringenin chalcone	$C_{15}H_{12}O_5$	272.253	8.518	Chalcones	"73692-50-9"	5067000	303800	392400	5591000	647200	292900
303	4-Methoxycinnamic acid	$C_{10}H_{10}O_3$	178	8.56	Phenylpropanoids	"830-09-1"	50250	16540	519800	38720	1088000	1480000
304	Butin	$C_{15}H_{12}O_5$	272.069	8.61	Flavanones	"492-14-8"	3180000	198900	279700	3401000	440900	211000
305	5,6-Dehydroginsenoside Rd	$C_{48}H_{80}O_{18}$	945.152	8.61	Terpenoids	"1268459-68-2"	13750	28850	6934	5526	63000	10290
306	Ginsenoside Rb1	$C_{54}H_{92}O_{23}$	1109.295	8.63	Terpenoids	"41753-43-9"	52090	14880	17064	47590	35530	53971
307	Naringenin	$C_{15}H_{12}O_5$	272.25	8.683	Flavanones	"480-41-1"	1140000	64380	92490	1209000	147700	83540
308	Apigenin	$C_{15}H_{10}O_5$	270.24	8.7	Flavones	"520-36-5"	396700	30440	28520	489700	74450	41720
309	Ethylparaben	$C_9H_{10}O_3$	166.17	8.703	Polyphenol	"120-47-8"	16160	29470	7311	34270	363300	8252
310	Genistein	$C_{15}H_{10}O_5$	270.24	8.72	Isoflavonoids	"446-72-0"	119400	1947	19436	95620	6499	51449
311	Methyl 4-hydroxycinnamate	$C_{10}H_{10}O_3$	178.18	8.76	Phenylpropanoids	"19367-38-5"	317600	119500	3151000	289200	6653000	7004000
312	Crocetin	$C_{20}H_{24}O_4$	328.408	8.79	Terpenoids	"27876-94-4"	172590	45780	74550	232147	15750	357800
313	Kaempferol	$C_{15}H_{10}O_6$	286.24	8.811	Flavonols	"520-18-3"	12560000	290100	2681000	13170000	2052000	3843000
314	7-O-Methyleriodictyol	$C_{16}H_{14}O_6$	302.079	8.86	Flavonoids	"51857-11-5"	89360	8616	15180	99050	24770	29630
315	Ginsenoside-Ro	$C_{48}H_{76}O_{19}$	957.119	8.86	Terpenoids	"34367-04-9"	143600	270100	46800	124200	865300	201000
316	Diosmetin	$C_{16}H_{12}O_6$	300.26	8.911	Flavones	"520-34-3"	556700	49230	39570	643800	75770	56620
317	Gardenoside	$C_{17}H_{24}O_{11}$	404.366	8.92	Terpenoids	"24512-62-7"	89125	15880	26170	105422	29694	140300
318	Isorhamnetin	$C_{16}H_{12}O_7$	316.262	8.969	Flavonols	"480-19-3"	606500	4657	66010	703000	24690	71630
319	Picropodophyllin	$C_{22}H_{22}O_8$	414.41	9.16	Lignans	"477-47-4"	6884	7386	6111	3755	2470	12966
320	4-Chloro-2-hydroxybenzoic acid, 4-chloro salicylic acid	$C_7H_5ClO_3$	172.57	9.167	Benzoic acids	"5106-98-9"	96405	11210	31850	120301	14480	120300
321	Steviolbioside	$C_{32}H_{50}O_{13}$	642.732	9.17	Terpenoids	"41093-60-1"	64460	17771	1425	90650	28720	19760
322	Ginsenoside Rd	$C_{48}H_{82}O_{18}$	947.154	9.33	Terpenoids	"52705-93-8"	6865	22110	5013	16978	9496	4825
323	Chikusetsusponin Iva	$C_{42}H_{66}O_{14}$	794.977	9.34	Terpenoids	"51415-02-2"	17080	2380	3192	21120	10120	12030
324	p-Coumaric acid ethyl ester	$C_{11}H_{12}O_3$	192.21	9.387	Phenylpropanoids	"7362-39-2"	10070	36353	7219	15130	123000	18630
325	Aurantio-obtusin	$C_{17}H_{14}O_7$	330.289	9.734	Quinones	"67979-25-3"	270200	23630	388100	306500	20690	971900
326	Ethyl ferulate	$C_{12}H_{14}O_4$	222.24	9.8	Phenylpropanoids	"4046-02-0"	2184	9348	3746	4696	28930	14520
327	Orcinol glucoside	$C_{13}H_{18}O_7$	286.28	9.914	Polyphenol	"21082-33-7"	864300	4264000	1909000	833600	6275000	3134000
